# Evaluation of pharmacogenomic testing to identify cytochrome P450 and SLCO1B1 enzymes and adverse drug events: A non-experimental observational research

**DOI:** 10.1097/MD.0000000000042031

**Published:** 2025-04-04

**Authors:** Tiffany Flowers-Moore, Alexandra R. Rapp, Jose H. Salazar, Rajkumar Rajendran

**Affiliations:** a Department of Clinical Laboratory Sciences, University of Texas Medical Branch, Galveston, TX; b Department of Pathology, University of Texas Medical Branch, Galveston, TX.

**Keywords:** cytochrome P450, drug–drug interactions, drug–gene interactions, PGx, pharmacogenomic testing, SLCO1B1

## Abstract

A laboratory-initiated preemptive and reactive cytochrome P450 and SLCO1B1 PGx testing protocol was evaluated in a private toxicology laboratory with the intent of identifying enzyme frequencies and associated adverse drug events. This study involved non-experimental observational research. During the retrospective medical chart review, patient demographics, statements of medical necessity, and PGx testing data were collected. Frequencies and percentages were calculated for the collected data, and statistical analysis was performed using Intellectus online software. A total of 192 PGx patient records from September 2019 to October 2021 were retrospectively reviewed. For patient demographics, men (n = 118; (61%)) were the majority gender identified among the patient population and Caucasians (n = 112; (58%)) followed by African Americans (n = 37; (19%)) were the most identified ancestry. The mean age of the patients was 69 (±9) years. CYP1A2 hyperinducers, followed by CYP3A5 poor metabolizers and CYP2B6 intermediate metabolizers, are the most encountered cytochrome P450 and SLCO1B1 enzymes. Regarding drug–gene interactions, 41 patients had 1 interaction, 29 had 2, and 31 had 3 or more interactions. For drug–drug interactions, 35 patients had 1 interaction, 15 had 2, and 30 had 3 or more interactions. Overall, 123 patients showed a minor or greater impact on drug–drug or drug–gene interactions. Overall, our study identified cytochrome P450 and SCLCO1B1 enzyme frequencies and patients experiencing actionable adverse drug events. By raising awareness of PGx test results through individualized clinician training, education, and interventions, these adverse events can be promptly identified and resolved.

## 1. Introduction

Pharmacogenomic testing (PGx) is the study of how genes affect a person’s response to medication. More than 50 cytochrome P450 genes have been identified in humans. However, CYP1A2, CYP2C9, CYP2C19, CYP2D6, CYP3A4, and CYP3A5 are the principal enzymes that metabolize 90 percent of drugs.^[1]^ Solute carrier organic anion transporter family member 1B1 (SLCO1B1) is a liver-specific transporter that is actively involved in active cellular influx of many endogenous and xenobiotic compounds.^[2]^ Advances in human genetic testing and a better understanding of drug metabolic pathways have led to the characterization of numerous gene mutations and discovery of functional pharmacogenetic polymorphisms. These variations in genetic polymorphism can be idiosyncratic, leading to unpredictable medication responses and potential activation of the immune system.^[3]^ Although only a small percentage of the genome differs between individuals, these differences can cause extensive changes in phenotypes, including drug metabolism response.^[4]^ Large population studies have shown that 95 percent of the population is estimated to carry at least 1 genetic variant that is discordant with at least 1 medication.^[5]^

Expression of cytochrome P450 enzymes may be influenced by many different factors and combinations of factors, including genetic polymorphisms, age, sex, induction of xenobiotics, hormones, and different disease states.^[6]^ Multiallelic genetic polymorphisms play a major role in CYP2D6, CYP2C19, CYP2C9, CYP2B6, CYP3A5, and drug metabolism. These polymorphisms often lead to different and distinct drug-metabolizing phenotypes, including poor metabolizers, intermediate metabolizers, normal metabolizers, rapid metabolizers, ultra-rapid metabolizers, and hyperinducers. CYP1A2 and CYP3A4 are less influenced by polymorphisms and more influenced by xenobiotic induction, age, sex, ethnicity, inflammation, and different disease states.^[6]^ Regarding SLCO1B1, about 1 in 40 people have increased SLCO1B1 function, 3 in 10 have decreased function and 1 in 40 have poor SLCO1B1 function and is clinically utilized to assess patients for the risk of statin-associated muscle symptoms.^[7,8]^

Preemptive and reactive PGx testing are 2 approaches to evidence-based medication management that have recently been investigated.^[3]^ For the process of preemptive testing, drug response genes are tested as a large multigene panel to optimize a patient’s medication use by having genetic information available at the point of prescribing.^[9]^ Many payers, apprehensive of the large initial costs and limited knowledge of downstream benefits of preemptive testing, have been influential in the resistance to the implementation of this type of medical care. However, the Pittsburgh Medical Center, St. Jude Children’s Research Hospital, and Vanderbilt University Medical Center have recognized the value of pharmacogenomics and invested in large, population-scale, preemptive testing programs.^[10]^ Reactive PGx testing is based on an approach in which a patient receives genetic testing for a particular gene or gene after a clinical decision has been made to start the patient on medication with known PGx implications. Many reactive PGx tests are performed after documented therapeutic failure or acute drug reaction. Reactive PGx testing focuses on 1 or a few gene–drug pairs, and the cost of this type of testing is relatively low.^[11]^

Although many studies have shown that PGx testing can be used to refine the selection of a medication or dose, it is important to note that PGx results are predictive and do not guarantee that an outcome will occur with certainty.^[5]^ The implementation of PGx testing in routine clinical practice has largely been limited to physicians and mid-level practitioners as complementary care for pain management, warfarin therapy, and mental health treatments. Although genetic tests can predict the response to drug therapy for many commonly prescribed drugs, test use has historically been low in primary, secondary, and community care clinical practices. Lack of awareness of testing, clinicians not being comfortable interpreting and applying test results, and concern over the medical necessity and cost of testing are common barriers to implementation.^[12]^ By raising awareness of PGx testing through training and education, testing may be considered reasonable and necessary as an adjunct personalized medical decision-making tool once a clinician has narrowed treatment possibilities to a specific medication or a combination of medications. One example of a clinical medical decision-making tool that has been utilized in the PGx testing environment is the use of Pharmacogenomic Interaction Probability (PIP) scores and their role in role in predicting drug–gene polymorphisms and drug–drug interactions. A study with more than 36,000 patients demonstrated that adjusted PIP scores are comparable to PGx test regardless of the number of minimum genes tested. Furthermore, when used together, clinical decision-making tool, PIP scores and PGx testing form a practical alternative to universal preemptive testing.^[13]^ Multidisciplinary clinics that provide genotyping and related services can facilitate the integration of pharmacogenomics into routine clinical care and meet the needs of patients on a wider scale. Decisions to initiate, modify, stop, or withhold medication treatments can be made from PGx testing results, reducing the possibility of adverse drug events, and improving downstream patient management.

The overall objective of this study was to evaluate patients in a private toxicology laboratory for clinical indicators for cytochrome P450 and SLCO1B1 pharmacogenomic polymorphism testing and identify areas to reduce adverse drug events. The specific goals of this study were to evaluate patient records and physician orders to provide a review of the medical necessity for PGx testing, including the frequency of orders based on preemptive or reactionary medical necessity statements for each patient. In addition, we identified the frequency of cytochrome P450 and SLCO1B1 drug-metabolizing phenotypes and the frequency of actionable drug–drug and drug–gene interactions encountered in the studied patient population. Drug-metabolizing phenotypes were categorized as poor metabolizers, intermediate metabolizers, normal metabolizers, rapid metabolizers, ultra-rapid metabolizers, and hyperinducers. The interactions were categorized as minimal, minor, moderate, major, or contraindicated. The outcome of the study will provide details regarding pharmacogenetic testing patterns, identify adverse drug events, and the need for a multidisciplinary team of specialized professionals, including individuals from the laboratory, that can aid in appropriate laboratory test utilization and interpretation of results to decrease unnecessary healthcare costs and ultimately improve patient outcomes.

## 2. Methods/study design

**Figure 1.**
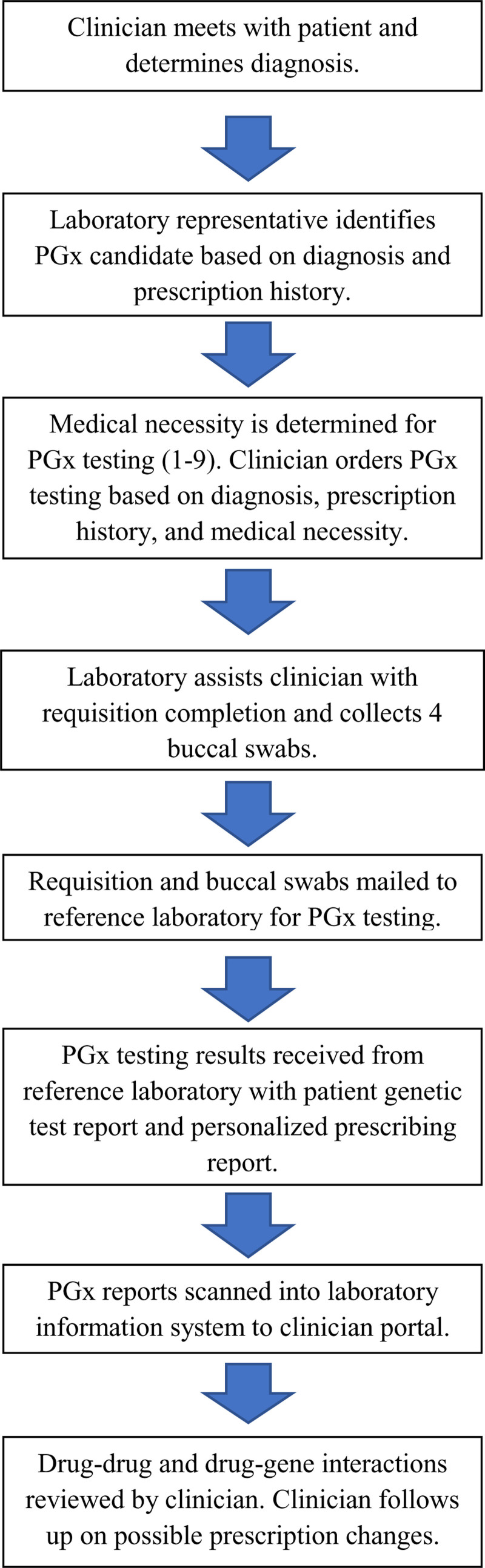
Laboratory-initiated pharmacogenomics (PGx) testing algorithm. Algorithm outlining sequential steps within the PGx testing process from patient diagnosis to test result interpretations.

The research design used in this study was non-experimental observational research. A retrospective chart review of the patients with PGx test orders from September 2019 to October 2021 was performed. Figure 1 illustrates the workflow of the PGx test ordering process from initial test order to laboratory personnel uploading results to clinic’s EMR for provider review. The study was conducted in urology, pain management, anesthesiology, and family medicine clinics that serve a diverse patient population in Texas. Data collected included patient demographics (age, sex, race), pharmacogenomic laboratory test results and interpretations (Genelex and YouScript® Clinical Decision Support Tool, Seattle), completed PGx statement of medical necessity, and ordering provider demographics collected from the Invitae, Go Rev, and Stratus DX reference laboratory information systems.

In the study’s location, PGx testing are send-out tests that are performed by a third-party company (Genelex) that includes the following pharmacogenomic tests including variants recommend by the Association of Molecular Pathology (AMP) PGx working group as part of their panel: CYP2D6, CYP2C19, CYP2C9, CYP3A4, CYP3A5, VKORC1, HLA-B*57:01, SLCO1B1, UGT1A1, TPMT, DPYD (DPD), IFNL3 (IL28B), NAT2, F2 (Factor II), F5 (Factor V) Leiden, MTHFR, CYP2B6, OPRM1, HTR2A, HTR2C, GRIK4, CYP1A2, COMT, ADRA2A, CYP4F2. In our study, only CYP2D6, CYP2C19, CYP2C9, CYP2B6, CYP3A5, CYP1A2, CYP3A4 and SLCO1B1 enzymes and phenotypic data were collected. The providers were identified as nurse practitioners, physician assistants, and medical doctors, who had the ability to order PGx testing and act on the final test results. For interaction impact which includes drug–drug interaction, drug–gene interactions and impact in patients, our study utilized the legend listed in the Genelex, YouScript Personalized Prescribing Report (Fig. S1, Supplemental Digital Content, http://links.lww.com/MD/O613). Frequencies and percentages were calculated for each nominal variable, including statements of medical necessity, cytochrome P450 and SLCO1B1 enzyme data, drug–drug and drug–gene interactions, and interaction impacts in patients. Statistical analyses were conducted using the online software Intellectus Statistics. The Institutional Review Board (IRB) designated the study as a quality assessment/improvement project; therefore, it did not require IRB approval or oversight.

## 3. Results

Overall, men comprised much of the patient population, with 118 (61%) participants. Caucasians represented 58% of the population with 112 participants. African Americans represented 19% of the population with 37 participants, Latin American/Caribbean patients accounted for 13% with 25 patients, 15 patients identified themselves as other, and 3 (2%) Asians participated, as seen in Table 1. The age of the studied patient population had an average of 69 years, with a standard deviation of ±9 years. The youngest patient who underwent PGx testing was 38 years old, while the oldest patient was 94 years old. Our study identified that the most used statement of medical necessity was “Test result will determine drug selection and/or dosage for a specific disease process” while “Patient has been using opioids with escalating doses and the result has been a subtherapeutic response” was chosen the least. The list of medical necessity statements and the frequency of selection are listed in Table 2. Data regarding cytochrome P450 and SLCO1B1 enzymes and enzyme metabolism frequencies for our study’s population are listed in Table 3.

**Table 1:** Patient demographics.

Variable			n (n = 192)	*%*
Sex				
Male			118	61
Female			74	39
Ancestry				
Caucasian			112	58
African American			37	19
Latin America/Caribbean			25	13
Other			15	8
Asian			3	2

	Mean	SD	Min	Max
Age	69	±9	38	94

**Table 2:** Frequency of medical necessity statements chosen by clinicians.

Variable	n (n = 192)	*%*
Test will determine drug selection and/or dosage for a specific disease process*		
No	22	11.46
Yes	170	88.54
Patient has been using opioids with escalating doses and the result has been a subtherapeutic response**		
No	190	98.96
Yes	2	1.04
Patient has been taking medications and is not reporting the outcomes anticipated at this point		
No	181	94.27
Yes	11	5.73
The patient is polypharmacy, and this may also be contributing to a subtherapeutic response		
No	177	92.19
Yes	15	7.81
Patient has tried various opioids and the result has been a subtherapeutic response		
No	182	94.79
Yes	10	5.21
Patient is taking a certain psychiatric or neurological medication		
No	153	79.69
Yes	39	20.31
Patient is taking antithrombotic medications		
No	156	81.25
Yes	36	18.75
Patient is taking cardiovascular medication		
No	121	63.02
Yes	71	36.98
Other		
No	177	92.19
Yes	15	7.81

Due to rounding, percentages may not equal 100%.

* Most frequently chosen.

** Least frequently chosen.

**Table 3:** Enzyme and metabolism data.

Variable	n (n = 192)	*%*
CYP2D6		
Normal metabolizer	95	49.48
Intermediate metabolizer	78	40.62
Poor metabolizer	12	6.25
Ultra-rapid metabolizer	7	3.65
CYP3A4		
Normal metabolizer	129	67.19
Intermediate metabolizer	63	32.81
CYP1A2		
Hyperinducer	136	70.83
Normal metabolizer	54	28.12
Intermediate metabolizer	1	0.52
Poor metabolizer	1	0.52
CYP2C19		
Normal metabolizer	86	44.79
Rapid metabolizer	54	28.12
Intermediate metabolizer	35	18.23
Poor metabolizer	13	6.77
Ultra-rapid metabolizer	4	2.08
CYP3A5		
Poor metabolizer	127	66.15
Intermediate metabolizer	53	27.60
Normal metabolizer	12	6.25
CYP2C9		
Normal metabolizer	134	69.79
Intermediate metabolizer	55	28.65
Poor metabolizer	3	1.56
CYP2B6		
Intermediate metabolizer	85	44.27
Normal metabolizer	78	40.62
Poor metabolizer	29	15.10
SLCO1B1		
(521TT) normal function	140	72.92
(521TC) decreased function	50	26.04
(521CC) poor function	2	1.04

Due to rounding errors, percentages may not equal 100%.

**Figure 2.**
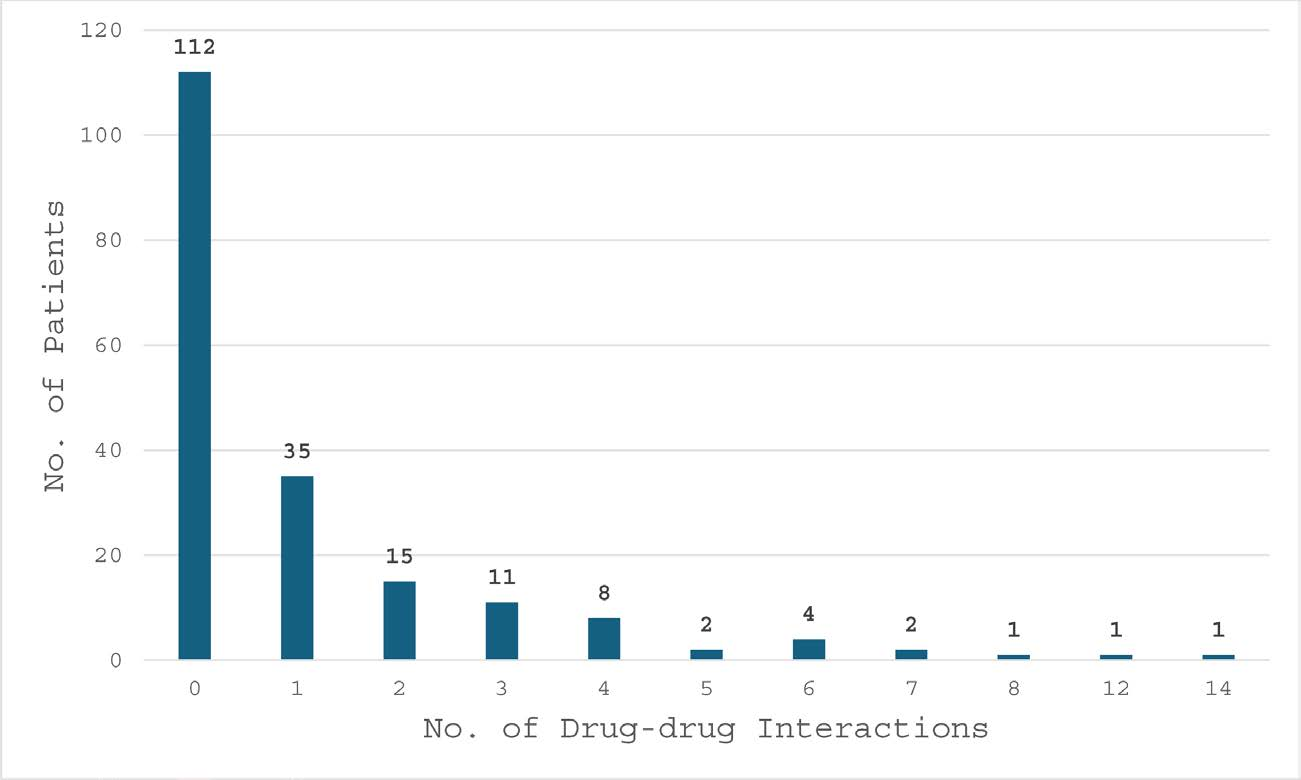
Drug–drug interactions per patient. Bar graph illustrating the number of drug–drug interactions per patient, highlighting the frequency and distribution of interactions across the study population.

**Figure 3.**
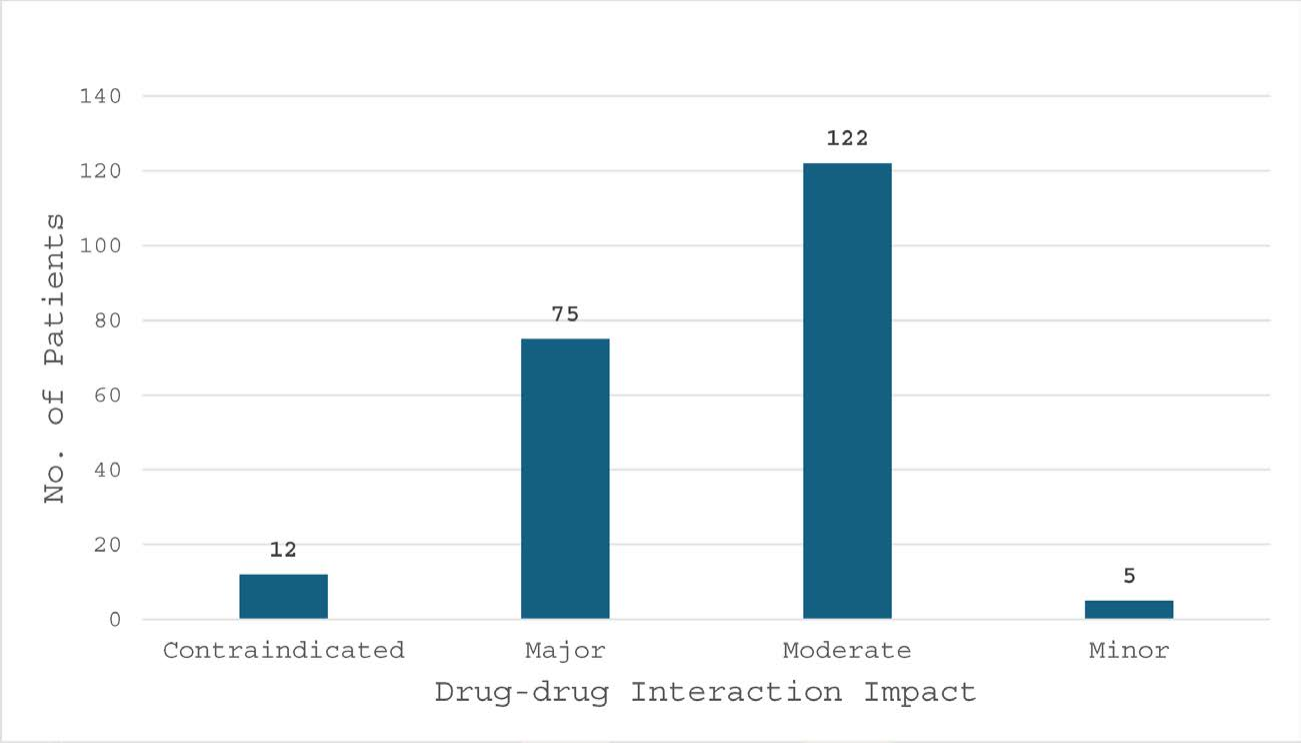
Total drug–drug interaction impacts. Bar graph depicting the impact of drug–drug interactions, showcasing the frequency and severity of interactions within the study population. *Contraindicated: interaction is contraindicated due to potential risk of severe or life-threatening reaction, risk outweighs benefits. *Major: interaction may result in severe clinical effects, risk outweighs benefit. *Moderate: modify therapy, interaction result in substantial clinical effects, intervention required to minimize risk. *Minor: Monitor therapy, interaction result in minor clinical effects, benefit generally outweighs risk. *Sourced from YouScript Personalized Prescribing Report (Fig. S1, Supplemental Digital Content, http://links.lww.com/MD/O613).

Regarding drug–drug interactions, out of 192 patients reviewed, 112 had 0 drug–drug interactions, 35 patients had at least 1 drug–drug interaction, 15 had 2 interactions, 11 had 3 interactions, 8 had 4 interactions, and the rest of the patients had a range of 5 to 14 drug–drug interactions, as shown in Figure 2. In the 80 patients that were identified to have had a drug–drug interactions, there were a total of 216 drug–drug interactions. Drug–drug interactions were stratified based on the interaction impact legend (YouScript Personalized Prescribing Report), 12 interactions were contraindicated, 75 were major, 122 moderate and 2 minor interactions as show in Figure 3.

**Figure 4.**
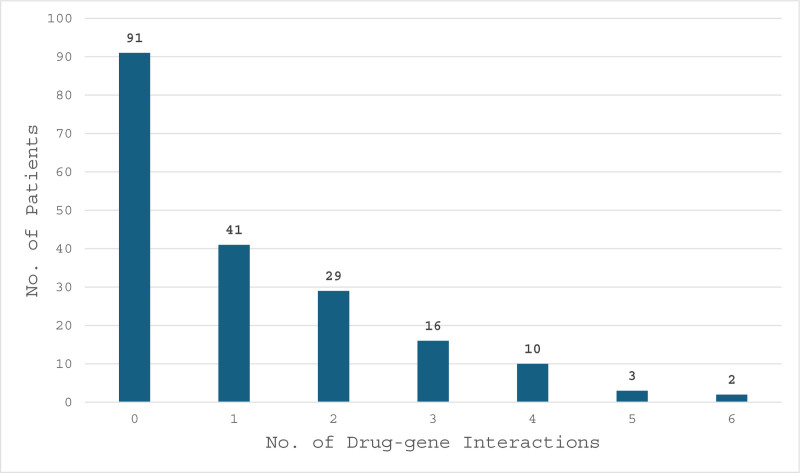
Drug–gene interactions per patient. Bar graph illustrating the number of drug–gene interactions per patient, highlighting the frequency and distribution of interactions across the study population.

**Figure 5.**
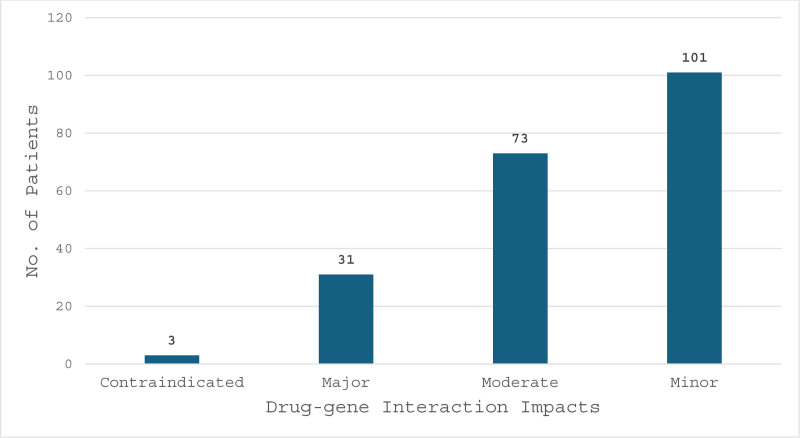
Total drug–gene interaction impacts. Bar graph depicting the impact of drug–gene interactions, showcasing the frequency and severity of interactions within the study population. *Contraindicated: interaction is contraindicated due to potential risk of severe or life-threatening reaction, risk outweighs benefits. *Major: interaction may result in severe clinical effects, risk outweighs benefit. *Moderate: Modify therapy, interaction result in substantial clinical effects, intervention required to minimize risk. *Minor: Monitor therapy, interaction result in minor clinical effects, benefit generally outweighs risk. *Sourced from YouScript Personalized Prescribing Report (Fig. S1, Supplemental Digital Content, http://links.lww.com/MD/O613).

For drug–gene interactions, our study identified that out of 192 patients, 91 patients had 0 drug–gene interactions, while 41 had at least 1 interaction, followed by 29 patients with 2 interactions, 16 with 3, 10 with 4, 3 with 5, and 2 with 6 drug–gene interactions, as shown in Figure 4. In the 101 patients that were identified to have had a drug–gene interactions, there were a total of 208 drug–gene interactions. Again, drug–gene interactions were stratified based on the interaction impact legend, 3 interactions were contraindicated, 31 were categorized as major, 73 moderate and 101 minor interactions, as shown in Figure 5.

## 4. Discussion

Until recently, physician-ordered pharmacogenetic testing was primarily used as complementary care for pain management, warfarin therapy, cancer care, and mental health treatments. Although genetic testing can predict the response to drug therapy for commonly used drugs, its use has been low. The primary objective of this study was to identify the frequencies of cytochrome P450 and SLCO1B1 enzyme data and identify areas to reduce adverse drug events. By raising awareness of PGx testing through individualized clinician training and education, it may be clinically useful to implement laboratory-initiated PGx testing, and interpretations provided by experts and/or interprofessional teams that includes a Doctorate in Clinical Laboratory Scientists (DCLS) in primary care, secondary care, and community practice.

In this study, we retrospectively reviewed 192 PGx patient records using the clinic’s EMR. Overall, men comprised much of the patient population, with 118 (61%) participants. Regarding ancestry, there were 112 Caucasians (58%), 37 African Americans (19%), 25 Latin American/Caribbean (13%), 3 Asians (3%), and 15 other (%). The average age of the patient population was 69 (±9) years; 38 was the youngest, while 94 was the oldest. It was observed that our study’s patient demographics closely resembled those of a doctorate in pharmacy-initiated study, differing only in that ours was led by a DCLS.^[14]^ The pharmacy-led study included 97 males and 103 females, with a mean age of 62 ± 11 years. Among its 92 participants, 92% were Caucasian, 3% Asian, 2.5% mixed descent, 1.5% Arabic, and 0.5% Somali.

In our study, of the 9 medical necessity statements provided to the clinicians, the least commonly selected was “Patient has been using opioids with escalating doses and the result has been a subtherapeutic response.” Only 2 (1%) patients were given this reason for testing. The most used medical necessity statement selected was “Test result will determine drug selection and/or dosage for a specific disease process.” Of the 192 patients who were PGx tested, 170 (88.5%) were given this statement as a basis for testing. These findings indicate that clinicians acknowledge the need for caution when prescribing medications with clinically significant drug–gene interactions, particularly CYP450 inhibitors or inducers.

The medical necessity statements that were provided on the patient test requisition were designed to provide clinicians with a standardized language and justification of appropriate testing for potential insurance reimbursement. Despite the growing use of PGx testing in healthcare, insurance coverage remains limited to cases where a clinician deems a medication with a known drug–gene interaction medically necessary. Proper documentation is essential for billing, reimbursement, and recordkeeping. In 2012, the American Medical Association assigned unique CPT codes to pharmacogenetic tests known to detect genetic variants associated with specific drugs; these codes were implemented in 2013 and updated in 2018.^[15]^ Although standardized CPT codes and ICD-10 codes have been designated for the purposes of PGx billing after testing has been completed, the current literature lacks clear guidelines on establishing medical necessity for PGx testing before it is performed.

Regarding the frequencies of cytochrome P450 and SLCO1B1 drug-metabolizing phenotypes, the patient population in this study exhibited predominantly normal metabolizer status for the CYP2D6, CYP3A4, CYP2C19, CYP2C9, and SLCO1B1 enzymes. However, CYP1A2 hyperinducers were most frequently encountered, as were CYP3A5 poor metabolizers and CYP2B6 intermediate metabolizers.

CYP2D6 is 1 of the most polymorphic human genes responsible for the metabolism or bioactivation of approximately 25% of drugs, including antidepressants, antipsychotics, analgesics, and anticancer agents.^[4]^ With considerable variability among ethnic groups, the translation of this highly complex, polymorphic CYP2D6 genotype data into predictive phenotypes can be complicated, and some genotypes may fall somewhere between the established categories of functional metabolizer status.^[16]^ The patient population for this study reflected the proven variability and showed an equal division of normal metabolizers (49.5%) to the combination of intermediate metabolizers, poor metabolizers, and ultra-rapid metabolizers at 50.5%.

The CYP3A family of enzymes is 1 of the most important groups of drug-metabolizing enzymes. CYP3A4 comprises over 60% of all hepatic and intestinal cytochromes and catalyzes the biotransformation of over 50% of the most commonly used drugs.^[17]^ Possible regulatory factors may include ethnicity, age, sex, disease state, hepatocellular function, nutrition, circulating hormones, inflammation, drug–drug interactions, consumption of grapefruit juice, and herbal preparations.^[6]^ During the testing of this patient population, CYP3A4 enzymes were measured as normal metabolizers for 67% of the population. The remaining 33% of the patients were intermediate metabolizers. A variety of factors, including advanced age of the tested population, disease state, medication use, and ethnicity, may account for the deviation from the normal metabolizer status.

CYP2C19 genetic testing is often used to identify patients who are unlikely to respond to treatment with the antiplatelet prodrug clopidogrel. The frequency of loss-of-function mutations in CYP2C19 ranges from 2% to 5% in Caucasians and African Americans, and from 13% to 23% in people of Asian ancestry.^[18]^ In 2010, the clopidogrel package insert was updated to include a boxed warning about reduced drug effectiveness in CYP2C19 poor metabolizers. It also advised clinicians on the potential interaction between clopidogrel and proton pump inhibitors (PPIs), as most PPIs are metabolized by CYP2C19.^[19]^ Interestingly, the patient population in this study revealed a 2% poor metabolizer status, coinciding with the previous population statistics. Several clinical studies have confirmed that CYP2C19 poor metabolizers have a significantly lower anticoagulation effect of clopidogrel, which has been associated with an increased risk of major adverse cardiovascular events.^[6]^ Conversely, this study revealed that approximately 35% of the population are rapid and ultra-rapid metabolizers, indicating that there are 2 copies of the CYP2C19 gene, resulting in increased CYP2C19 activity. Clinicians should consider Food and Drug Administration (FDA) warnings and adjust prescriptions and dosages with caution once the metabolizer status has been established.

Our study revealed a group of 70% normal metabolizers and a combination of 30% intermediate and poor metabolizers of CYP2C9. The gene encoding the CYP2C9 enzyme carries numerous inherited polymorphisms and is expressed at similar or even higher protein levels than CYP3A4.^[6]^ Currently, more than 100 drugs have been identified as substrates of CYP2C9. CYP2C9, along with VKORC1, plays a key role in regulating warfarin’s anticoagulant activity. Patients who are CYP2C9 intermediate or poor metabolizers have been associated with complications during warfarin initiation and prolonged hospitalization. Phenotype frequency studies of CYP2C9 polymorphisms indicate that normal metabolizers are most prevalent among Asians (96.5%), followed by Africans (87%). In contrast, Caucasians exhibit a lower prevalence of normal metabolizers (65.3%), with 34.7% classified as intermediate or poor metabolizers.^[20]^

Both genetic and environmental factors influence the activity of CYP1A2. This enzyme is more active in men than in women, is highly expressed in the liver, and is highly inducible in the liver, lung, pancreas, gastrointestinal tract, and brain.^[21]^ Ethnic differences have indicated that lower CYP1A2 activity has been measured in Asian and African populations than in Caucasians. CYP1A2 is responsible for more than 95% of the primary metabolism of caffeine and is highly inducible by cigarette smoking, cruciferous vegetables, charcoal grilled meats, heavy exercise, and PPIs.^[22]^ Although this study did not collect data on patient smoking status, caffeine consumption, eating, or other lifestyle habits, hyperinducer activity represented almost 71% of the patient population. 86 men and 50 women were classified as hypermetabolizers. Normal metabolizers represented 28% of the tested population, with very few poor (0.5%) or intermediate (0.5%) metabolizers.

In this study, Caucasians accounted for 58.3% of the patient population, with a high number (66%) of poor metabolizers of CYP3A5. Poor metabolizers and individuals with 2 nonfunctional copies of CYP3A4 alleles are rare. However, CYP3A5 poor metabolizers are common, particularly in Caucasian population.^[4]^

CYP2B6 genes exhibit high levels of interpatient genetic variability between and within ethnicities. Genetic polymorphisms, combined with environmental factors and gene regulation, are the major factors that affect variability in CYP2B6 expression and function.^[23]^ CYP2B6 is highly inducible by medications and xenobiotics. Age, sex, and disease condition are other confounders of CYP2B6 differential expression and function.^[24]^ This study indicated almost equal populations of normal and intermediate metabolizers, at 44% and 40%, respectively.

Although SLCO1B1is not a gene from the cytochrome P450 system, evidence clearly indicates that polymorphisms in the SLCO1B1 genotype affect statin pharmacokinetics and may increase the risk of myopathy in some patients. Senior age, comorbidities, and polypharmacy may contribute to the increased risk of myopathy. 27% of the studied patient population showed reduced or poorly functioning SLCO1B1. In 2014, clinical pharmacogenetics implementation consortium recommended a lower dose of simvastatin or an alternative statin as well as the potential benefit of routine creatine kinase investigation for patients with specific reduced-function alleles.^[25]^

Lastly, our study identified the frequencies of actionable drug–drug and drug–gene interactions in the studied patient population. Of the 192 patients tested, 112 (58.3%) showed no drug–drug interactions, 35 patients had 1 interaction, 15 had 2 interactions, 11 had 3 interactions, 8 had 4 interactions, and the remaining patients had a range of 5 to 14 drug–drug interactions. In total, 214 drug–drug interactions were distributed across 80 patients, of which 97.6% (209) interactions were scored as moderate or greater, while only 2.3% (5) were considered minor that could present with minimal clinical effects. Regarding drug–drug interactions in patients, moderate interactions were defined as impacts that have the possibility of substantial clinical effects. Interventions are frequently required to minimize the risk of moderate interactions. Examples of interventions include dose adjustments or the prescription of alternative medications. Major drug–drug interactions can result in severe clinical effects, and the associated risks outweigh the benefits. Contraindicated interactions pose a potential risk of severe or life-threatening reactions. These types of drug–drug combinations should be avoided.

In our study, 91 out of 192 patients had no drug–gene interactions, 41 patients had 1, 29 patients had 2 interactions, and 31 patients had ≥3 drug–gene interactions. Of the 208 drug–gene interaction impacts noted, minor impacts were the most encountered in this category, with 101 (49%) total interactions. There were 73 (35%) moderate, 31 (15%) major, and 3 (1%) contraindicated interactions. In total, 107 (51%) drug–gene interaction impacts were moderate or greater.

Due to the limitation within the clinic’s EMR and IRB restriction, our study was not able to identify whether an action was taken by the clinician. Another limitation of our study was that the primary objective was to collect and analyze only phenotypic data and identify drug–drug/drug–gene interactions, prompting the need for a geneticist or experts trained in pharmacogenomics, such a DCLS, who can guide clinicians with evidence-based recommendations to improve patient outcomes. A potential solution could be the implementation of Diagnostic Management Teams (DMTs) discussing pharmacogenomic testing, including actionable drug–drug and/or drug–gene interactions, to improve patient outcomes. A DMT provides a patient-specific, expert-driven narrative interpretation of the results, following a review of the patient’s past medical history, laboratory test results, and medications (clinical context) and inserts it into the patient’s medical record for provider review and action.^[26]^ Several studies showed effectiveness of using a DMT including avoidance of adverse clinical consequences, shortened length of stay, lower supply costs for patients and better patient outcomes when all the testing and interpretations were directed by a team of experts.^[27,28]^

Regarding decreasing adverse drug events, evidence show that implementation of interventions such as PGx test result interpretations, clinical decision systems and other resources that guide provider clinical decision-making can lead to improvement in patient care. For example, an established clinical decision support system provided pharmacogenomic results based on traffic light alerts, which were considered genomically favorable; yellow and red were associated with an increased risk of adverse reactions or non-response. Result of the study showed the total number of drug changes in response to potentially actionable alerts was 60 prescription changes out of 405 and independent of other potential prescribing mediators, medications with high pharmacogenomic risk changed significantly more often than those lacking pharmacogenomic information. No high-risk medications were prescribed during the study when physicians consulted the clinical decision support tool provided with the test results.^[29]^

Another study tested 205 patients (outpatients of cardiology, primary care, and internal medicine clinics) in the treatment arm of a prospective, observational cohort study where CYP2C9, CYP2D6, CYP2C19, CYP3A4, CYP3A5, and VKORC1 genes were panel-tested. The study reported that there was an average of 2 recommendations per patient, and 46% of the 381 test recommendations were followed and the study concluded that the PGx patients tested and treated according to the personalized report had a significant decrease in hospitalizations and emergency room visits, resulting in a potential cost estimated at a mean of $218 in the tested group. 95 of the clinical providers involved in the study, 95% considered the test helpful^[30]^

Our study had several limitations, the primary setting of which was a private clinical toxicology laboratory. The clinician and patient sample sizes may have been small, leading to the possibility of sample bias. The practicing clinician specialties included in the study were primarily pain management, urology, anesthesiology, and family medicine clinics, which may impose limitations on generalizability for all PGx testing and interpretations.

## 5. Conclusion

Overall, our study revealed several actionable drug–drug and drug–gene interactions in patients with PGx test results. By raising awareness of PGx testing through individualized physician training and education specifically in medical necessity statements as decision-making framework for the identification of PGx candidates and cytochrome P450 and SLCO1B1 drug phenotypes, PGx testing may be feasible and clinically useful in primary care, secondary care, and community practices to improve patient outcomes.

## Acknowledgments

The author would like to thank the entire Clinical Laboratory Sciences department and the department of Pathology at University of Texas Medical Branch in Galveston, Texas for their continued support and dedication.

## Author contributions

**Conceptualization:** Tiffany Flowers-Moore, Alexandra R. Rapp, Jose H. Salazar, Rajkumar Rajendran.

**Data curation:** Tiffany Flowers-Moore.

**Formal analysis:** Tiffany Flowers-Moore.

**Methodology:** Tiffany Flowers-Moore, Alexandra R. Rapp, Jose H. Salazar, Rajkumar Rajendran.

**Supervision:** Rajkumar Rajendran.

**Writing – original draft:** Tiffany Flowers-Moore, Rajkumar Rajendran.

**Writing – review & editing:** Tiffany Flowers-Moore, Alexandra R. Rapp, Jose H. Salazar, Rajkumar Rajendran.

## Supplementary Material


