# Supplementary material for: Evaluation of pharmacogenomic testing to identify cytochrome P450 and SLCO1B1 enzymes and adverse drug events: A non-experimental observational research
Source: Medicine (Baltimore). 2025 Apr 4;104(14):e42031. doi: 10.1097/MD.0000000000042031 (PMC11977719; doi:10.1097/MD.0000000000042031)
Supplement: Supplementary file 1 [file medi-104-e42031-s001.docx]

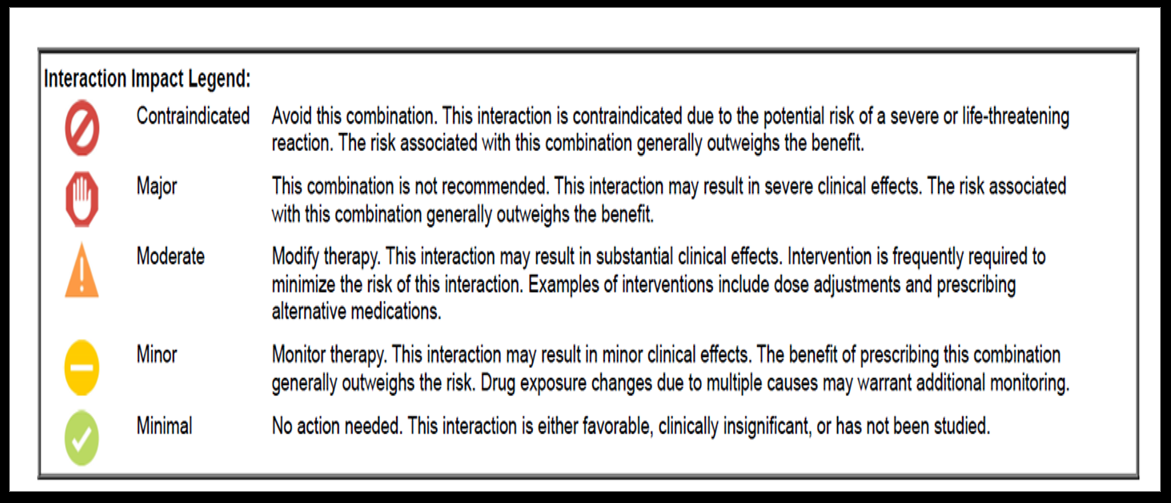


**Supplementary Figure 1.** Drug-drug and drug-gene interaction impact legend. Figure lists interaction impact categories and definition of each category. Sourced from YouScript Personalized Prescribing Tool, https://youscript.com/
